# Application of Drone in Plastic Surgery

**DOI:** 10.29252/wjps.9.3.351

**Published:** 2020-09

**Authors:** Abhinav Aggarwal, Ravi Kumar Chittoria, Vinayak Chavan, Saurabh Gupta, Likhitha Reddy, Padma Lakshmi Bharathi Mohan, Imran Pathan, K. Shijina

**Affiliations:** Department Of plastic Surgery, Jawaharlal Institute of Postgraduate Medical Education and Research, Pondicherry, India

**Keywords:** Drone, Plastic surgery, Telemedicine, Tele-barrier nursing

**DEAR EDITOR**

Drone applications in medicine include disaster assessment,^1^ delivery of aid packages,^1^ vaccines,^1^ blood, rapid access to automated external defibrillators (AED) for patients in cardiac arrest,^2^ and rapid transport for organs for transplantation.^1^ Telemedicine is a field where drones have great possibilities, especially in the field of tele barrier nursing for an Intensive Care Unit (ICU) patient. ICU patients are gnerally immunocompromised and require intensive monitoring. This requires either constant presence of a doctor or repeated visits to the isolation ICU.^3^

Repeated visits can increase the risk of cross-infection through a doctor/health care professional that enters the room. Using a drone circumvents these issues and helps us monitoring the patient remotely, without any direct patient contact. Drones have already been used successfully in major disasters due to the ease to bypass road closures and rugged terrains without any flight crew.^3^ They have been ised in 2010 earthquatke in Hiati, 2012 in the United States, 2015 in islands on Vanuatu and for the 2015 earthquake in Nepal.^3^

The Médecins Sans Frontières/ Doctors without borders (MSF) used drones to transport dummy TB test samples in Papua New Guinea. The National Aeronautics and Space Administration (NASA) tested medical supply delivery to a small clinic in rural Virginia using a drone. The United Nations Children’s Fund (UNICEF) delivered human immunodeficiency viruses (HIV) kits in Malawi, Africa using a drone too.^3^ Drones in out of hospital arrest of the patient have been well documented. They have been used to transport AED’s in public place rapidly on call for ambulance in case of arrest.^2^

They have been shown to have a positive impact on survival following out of hospital cardiac arrests. Drones have the potential to be used for delivery of blood samples and other biological samples in a very cost effective way. The efficiency of the drone in transporting small goods is well documented and especially useful in hospitals where centralized sample delivery chute is unavailable.^4^^-^^6^

A prospective study was done in Plastic Surgery ICU of Jawaharlal Institute of Postgraduate Medical Education and Research (JIPMER) and JIPMER Tertiary Burns Center from December 2018 to January 2019. A nano-drone (IZI JX 1601HW RTF Mini WiFi FPV with 720P Camera Altitude Mode Foldable Arm RC Drone Quadcopter - 2.0MP White with weight of 191 g) was used to monitor the ICU patients that operated with the National Knowledge Network (NKN) in JIPMER (Figure 1). 

**Fig. 1:**
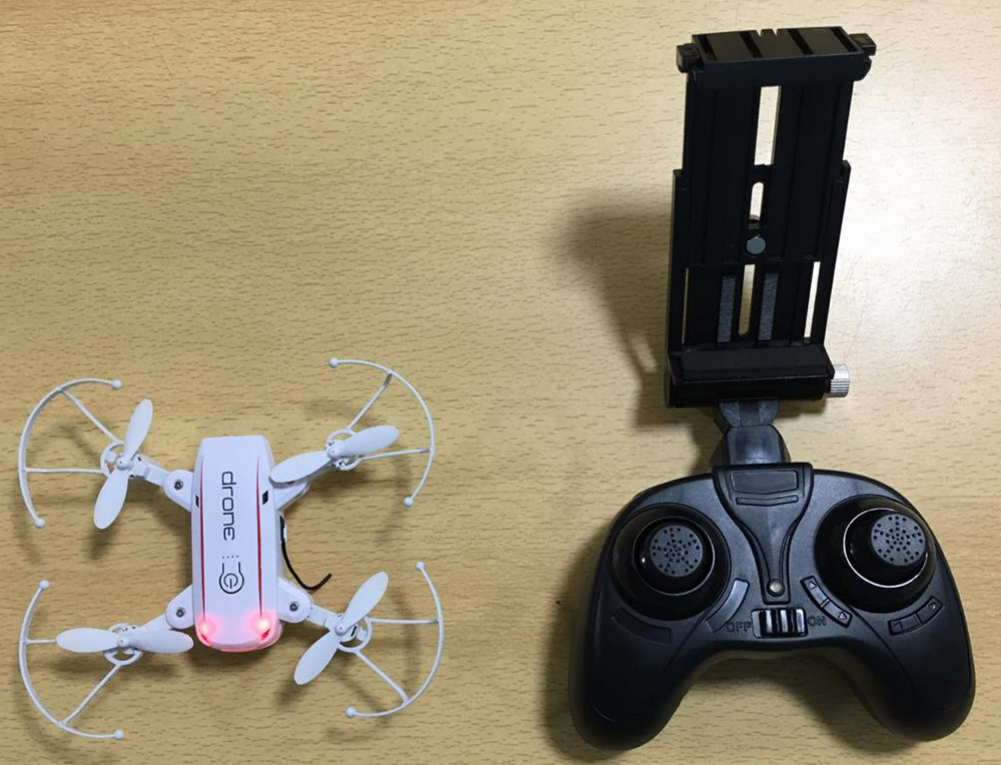
Nano-drone with console

The drone’s camera was 2 megapixel HD, which could be adjusted manually. It also had a console which could be used to control the drone as well as a rechargeable battery. The phone/monitor could be attached to the console. The flight range of the drone was 30 meters. The doctor who wanted to monitor the patient used the console to direct the drone into the patient’s room to view the patient’s vitals in the monitor, and only if required the doctor went inside to intervene (Figures 2-4). The drones were operated by six surgeons to monitor the patients in the ICU. One day basic training about the drain operation and functions was given by a trained executive. Feedback was taken from the person using the drone, and all six found the experience to be extremely satisfactory (Figure 5). 

**Fig. 2:**
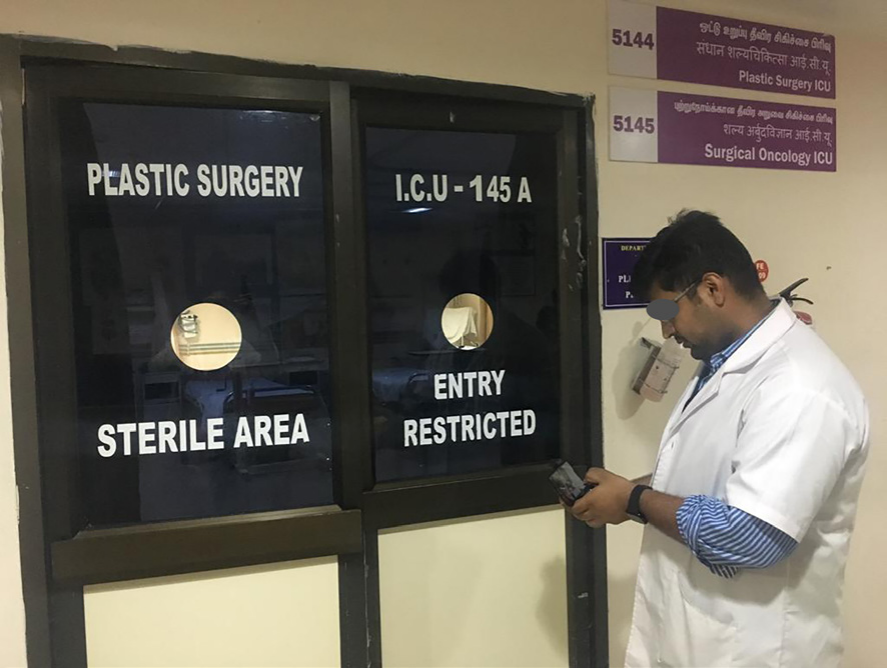
Drone being operated by the doctor outside the ICU

**Fig. 3:**
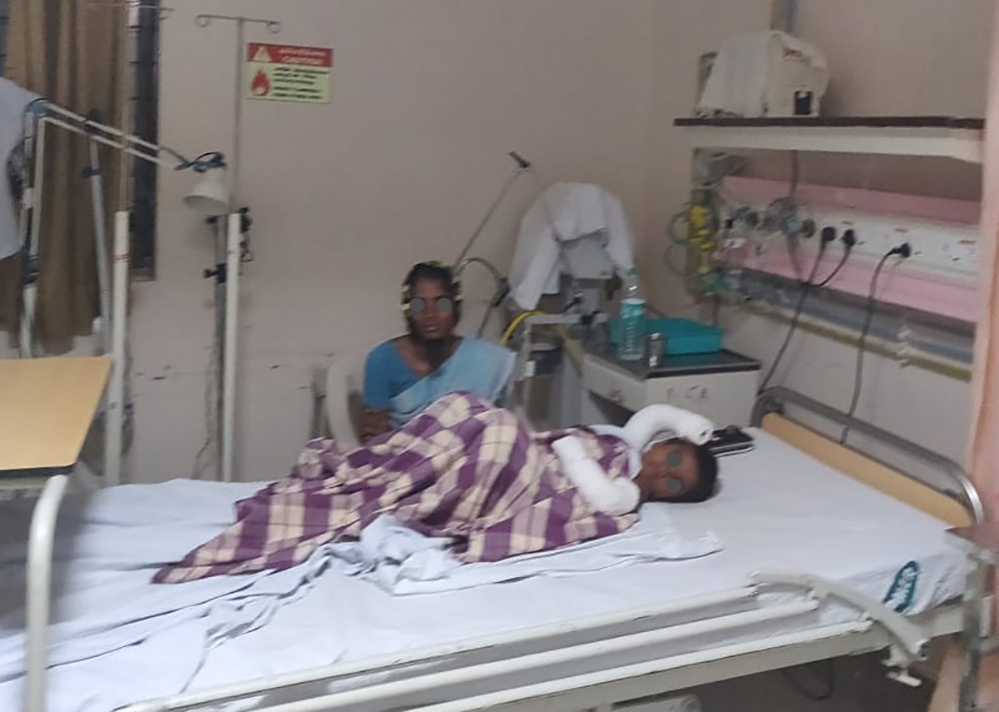
Drone monitoring a post-operative patient in the ICU

**Fig. 4:**
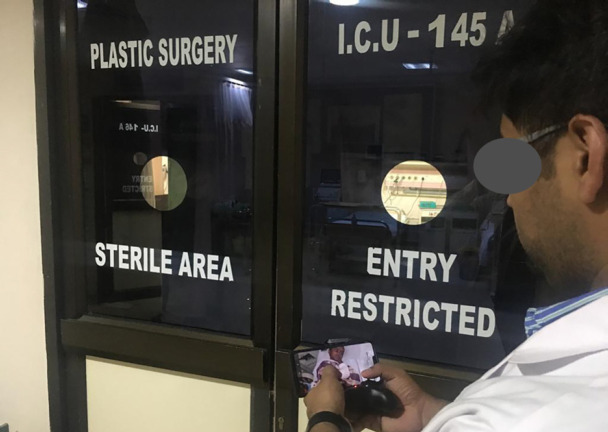
Patient being monitored outside the ICU using the drone

**Fig. 5:**
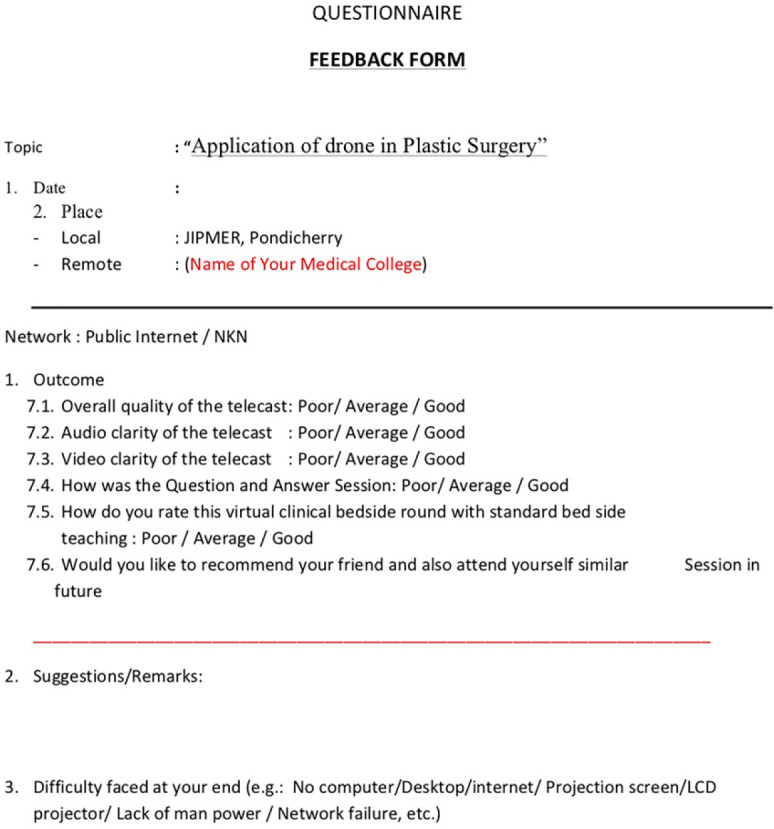
Feedback form

The term unmanned aerial vehicle (UAV) was the initial term used for a drone in the 1980s. Drones are under regulatory bodies like the Federal Aviation administration (FAA) in the USA. In India, The Directorate General of Civil Aviation (DGCA) are the regulatory body. They have classified into the drones have been divided into five categories including (i) Nano: Less than or equal to 250 grams, (ii) Micro: From 250 grams to 2 kg, (iii) Small: From 2 kg to 25 kg, (iv) Medium: From 25 kg to 150 kg, and (v) Large: Greater than 150 kg.

Use of drones for tele-barrier nursing is a novel one and has not become popular yet. In terms of surveillance, drones have been used in Sweden as a monitoring device for individuals in beaches. Claesson *et al.* conducted a study comparing time saved to locate a drowning individual in the water using drones and concluded that 3 mins and 38 secs were saved using drones. The study is an interesting insight on the topic and has prospective uses in saving manpower and making monitoring in icus much more efficient.

The limiting factor to usage of drones is the Federal Aviation Administration (FAA) legislations. According to FAA, drones must be always in line of sight of the pilot and cannot exceed an altitude of 400 feet and go faster than 100 miles per hour. These legistations restrice the usage of drones for most of the indications discussed above. Use of nano-drones for indoor tele-monitoring; however, is free from these legislations but runs a risk of crashing into obstacles and equipment. Furthur effort and studies into the topic may be required to felicitate our claims. Drones have a promising feature in the field of medicine. Tele-medicine is a novel field and full of innovations. The use of drone is a step in the same direction and can be used to make monitoring of critical patients more cost effective and easier in addition to the existing medical uses of the drones.

## CONFLICT OF INTEREST

The authors declare no conflict of interest.
